# Rapid insecticide resistance bioassays for three major urban insects in Taiwan

**DOI:** 10.1186/s13071-023-06055-x

**Published:** 2023-12-02

**Authors:** Hsiu-Hua Pai, Chun-Yung Chang, Kai-Chen Lin, Err-Lieh Hsu

**Affiliations:** 1 Department of Kinesiology, Health, and Leisure Studies, National University of Kaohsiung, Kaohsiung, Taiwan (ROC); 2 Department of Entomology, National Taiwan University, Taipei, Taiwan (ROC)

**Keywords:** Bioassay, Discriminating dose, Discriminating concentration, Susceptibility, Insecticide resistance, House flies, Cockroaches, Mosquitoes

## Abstract

**Background:**

Taiwan’s warm and humid climate and dense population provide a suitable environment for the breeding of pests. The three major urban insects in Taiwan are house flies, cockroaches, and mosquitoes. In cases where a disease outbreak or high pest density necessitates chemical control, selecting the most effective insecticide is crucial. The resistance of pests to the selected environmental insecticide must be rapidly assessed to achieve effective chemical control and reduce environmental pollution.

**Methods:**

In this study, we evaluated the resistance of various pests, namely, house flies (*Musca domestica* L.), cockroaches (*Blattella germanica* L. and *Periplaneta americana*), and mosquitoes (*Aedes aegypti* and *Ae. albopictus*) against 10 commonly used insecticides. Rapid insecticide resistance bioassays were performed using discriminating doses or concentrations of the active ingredients of insecticides.

**Results:**

Five field strains of *M. domestica* (L.) are resistant to all 10 commonly used insecticides and exhibit cross- and multiple resistance to four types of pyrethroids and three types of organophosphates, propoxur, fipronil, and imidacloprid. None of the five field strains of *P. americana* are resistant to any of the tested insecticides, and only one strain of *B. germanica* (L.) is resistant to permethrin. One strain of *Ae. albopictus* is resistant to pirimiphos-methyl, whereas five strains of *Ae. aegypti* exhibit multiple resistance to pyrethroids, organophosphates, and other insecticides.

**Conclusions:**

In the event of a disease outbreak or high pest density, rapid insecticide resistance bioassays may be performed using discriminating doses or concentrations to achieve precise and effective chemical control, reduce environmental pollution, and increase control efficacy.

**Graphical Abstract:**

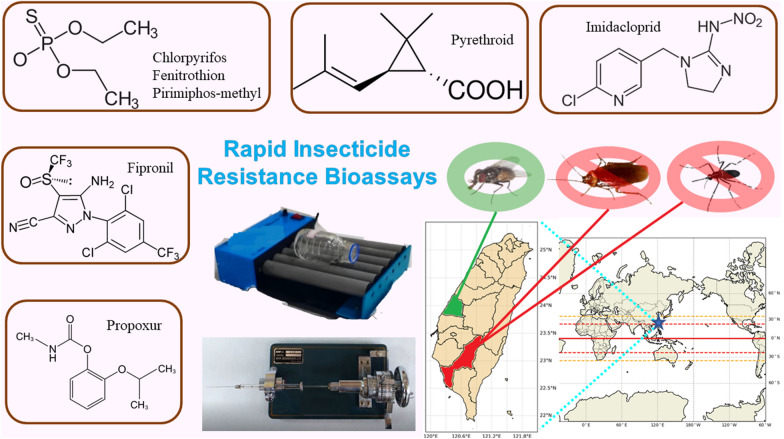

## Background

Taiwan’s warm and humid climate and dense population offer a suitable environment for the breeding of house flies, cockroaches, and mosquitoes. The house fly (*Musca domestica* L.) is a ubiquitous urban pest that has a long-standing association with both humans and domesticated animals. House flies can adapt to diverse human environments, including houses, garbage dumps, animal shelters, and food storage and delivery facilities; they are found in both tropical and temperate climates in developed and developing countries [1].

Although the house fly is often regarded as a nuisance pest, it is a notorious vector for more than 100 human and animal diseases caused by antibiotic-resistant zoonotic pathogens [2]. House flies have emerged as a public health concern in the urban environment because of their mobility and feeding behavior and their role in disease transmission. House fly management typically involves the use of insecticides. However, house flies have developed resistance against multiple insecticides, including pyrethroids, neonicotinoids, organophosphates (OPs), carbamates, organochlorines, and cyromazine (triazine) [3–6]. These insect pests are resistant to the active ingredients of insecticides that are used worldwide and thus have been classified as the most resilient urban insect pests [7].

In Taiwan, house flies commonly breed in traditional markets, garbage dumps, livestock farms, and chicken farms. Between 2015 and 2018, an increasing number of house flies in Taiwan were observed to be resistant to deltamethrin, chlorpyrifos, propoxur, and imidacloprid. The insects exhibited cross-resistance against four pyrethroid insecticides (cypermethrin, tetramethrin, permethrin, and deltamethrin) and three organophosphorus insecticides (chlorpyrifos, fenitrothion, and pirimiphos-methyl). House flies have developed resistance against multiple insecticides, with varying mechanisms of action [8].

The common species of cockroaches in Taiwan are *Blattella germanica* (L.) and *Periplaneta americana* [9]. Cockroach infestation is associated with poor sanitation, particularly in and around food-handling facilities, and tends to be more common in areas with lower socioeconomic status. Such an infestation may lead to food contamination and damage because cockroaches can transmit human and animal pathogens. In addition, cockroach feces, saliva, and cast skins contain certain allergens, which can trigger allergic reactions and psychological distress in sensitive individuals [10]. Cockroaches are among the most problematic urban pests that can initiate asthmatic and allergic reactions in children [11].

Cockroach management primarily involves the application of insecticide, with insecticide baits being the most popular and efficient formulation [12]. Conventional cockroach control programs have relied on the use of spray formulations containing carbamates, organophosphorus, and pyrethroids, leading to high levels of insecticide resistance in many field populations of cockroaches [13, 14]. In Taiwan, the German cockroach (*B. germanica*) has been reported to exhibit resistance to pyrethroids, organophosphorus, and carbamate insecticides [15]. These cockroaches have developed resistance against various insecticides, thus rendering chemical control strategies ineffective.

Many species of mosquitoes carry and transmit human pathogens [16]. For example, *Anopheles* mosquitoes contribute to the transmission of malarial parasites (*Plasmodium* spp.), thus facilitating the spread of malaria, which is among the top causes of mortality worldwide. The number of malaria cases continually increased between 2020 and 2021; however, the rate of growth was slower than that observed in the period between 2019 and 2020. The estimated number of malaria cases worldwide reached 247 million in 2021 compared with 245 million in 2020 and 232 million in 2019 [16]. *Aedes* mosquitoes, such as *Aedes aegypti* and *Ae. albopictus*, can transmit the viruses responsible for dengue, yellow fever, and chikungunya. A modeling study indicated that out of an annual number of 390 million dengue virus infections, 96 million result in clinical manifestations [17]. Another study on the prevalence of dengue revealed that 3.5 billion individuals are at a risk of dengue virus infection [18]. Species belonging to the genus *Culex* transmit West Nile virus, St. Louis encephalitis virus, Japanese encephalitis virus, and avian malarial parasites, all of which impose a substantial burden on public health [19, 20].

Pyrethroid resistance in *Anopheles* mosquitoes is widespread in many African countries [21, 22]. In addition, *Culex* mosquitoes worldwide [23] and *Aedes* mosquitoes from Singapore, Thailand, Malaysia, Brazil, Mexico, and Colombia [24] have exhibited pyrethroid resistance. Historically, cases of mosquito-borne infectious diseases, such as malaria, filariasis, Japanese encephalitis, and dengue, have been reported in Taiwan. In 1965, the World Health Organization (WHO) declared Taiwan a malaria-free area; no case of filariasis has been reported after 1960. Japanese encephalitis cases are rare because of the availability of vaccines and the high rate of vaccination (> 95%). Currently, dengue is the only mosquito-borne infectious disease that causes occasional outbreaks in Taiwan. *Aedes aegypti* and *Ae. albopictus* are the vectors of dengue virus. The resistance of *Ae. aegypti* to permethrin is a key reason for dengue outbreak [25]; insecticide resistance hinders the complete elimination of virus-carrying mosquitoes before the onset of a disease outbreak.

Selecting the most suitable environmental pesticide is crucial in the event of an outbreak or high pest density that necessitates chemical control. A rapid analysis of pesticide resistance may help achieve effective chemical control and reduce environmental pollution.

## Methods

### Insects

#### Susceptible strains

The susceptible strain of *M. domestica* was provided by the Department of Entomology, National Taiwan University (Taipei, Taiwan) in 2004 and maintained in a laboratory without any chemical exposure for more than 150 generations. Susceptible strains of *B. germanica* and *P. americana* were obtained from the Department of Entomology, National Taiwan University, and established in 1986. Susceptible strains of *Ae. albopictus* and *Ae. aegypti* (Bora Bora) have been bred for over 15 years. The control group comprised susceptible strains not exposed to any insecticide.

#### Field strains

Monitoring insecticide resistance in insects typically involves selecting counties or cities where resistance has occurred, collecting large numbers of samples from five locations (east, west, south, north, and central areas) in 2020 and performing various assays and analyses. House flies were collected using a sweep net at various garbage dumps. Cockroaches were lured using roach traps [9], which were placed on the floor under beds, cupboards, wooden racks, and benches for 7 consecutive days. Mosquitoes were collected using ovitraps [25], which are black cylindrical jars with a water-wetted paper strip inside. Ovitraps were placed both indoors and outdoors at each household in the selected counties or cities for 7 days. If insufficient insects were available, or further tests were desirable, insects could be reared in the laboratory and the first generation was used.

#### Insect rearing

The temperature and light of rearing rooms for the three types of insects were automatically controlled. The temperature and relative humidity were maintained at 27 °C ± 2 °C and 70% ± 10%, respectively. The susceptible and field strains of the insects were reared in separate rooms to prevent contamination.

#### House flies

Adult house flies were housed in insect breeding cages (size: 32.5 cm × 32.5 cm × 32.5 cm; MegaView Science Education Services Co., Ltd., Taichung, Taiwan). Adult flies were provided with water (10% syrup) and food (sugar and powdered milk). Female house flies were allowed to lay eggs. Then, these eggs were transferred to a larval medium comprising rat feed in hot water at a 1:1 ratio (160 g of mouse feed added to 160 ml water). After the maturation of the larvae, approximately 1-cm-thick wood chips were placed on top of the medium. The pupae were collected from the layer of wood chips, sifted into a petri dish, and placed in a new cage. After approximately 5–7 days, the pupae emerged as adult flies [8].

#### Cockroaches

Cockroaches were reared in cylindrical plastic containers (diameter, 23 cm; height, 30 cm) containing circular paper rolls. To prevent the cockroaches from escaping, the 10-cm-wide upper edge of the breeding box was coated with Vaseline. Cockroach nymphs and adults were provided with sufficient dog food (Fu Shou Industrial Co., Ltd.) and deionized water [9].

#### Mosquitoes

To rear *Ae. albopictus* and *Ae. aegypti*, a sheet of egg paper with approximately 200 eggs was placed at the bottom of a hatching tray filled with 800 ml deionized water and 3 ml larval food (a mix of pig liver powder and rabbit feed powder at a 1:1 ratio). After a few hours, the eggs hatched and the egg paper was removed. The floating film on the water surface was removed daily, and the larvae were fed appropriately. After 7 days, the larvae began to pupate and were transferred to pupal cups. Approximately 400 pupae were placed in mosquito cages for eclosion. Adult mosquitoes were provided with 10% sugar water and placed in separate rooms according to their strain. After 4–7 days since the emergence of adult mosquitoes, female mosquitoes were allowed to suck blood from mice placed in a blood-feeding device within the mosquito cage during the period between 10 a.m. morning and 5 p.m. evening. An egg-laying nonwoven cloth was placed along the cage’s edge with 20 ml water. After 4 days, the egg-laying paper was collected, dried, and stored in an airtight zipper bag [25].

### Insecticides

Ten technical-grade insecticides were obtained and diluted to one-fifth of their concentrations by using analytical grade acetone. The insecticides included pyrethroids (Aerolead International Ltd.; cypermethrin [92%], tetramethrin [92%], permethrin [92%], and deltamethrin [98%], OPs (chlorpyrifos [98%; Aerolead International Ltd.], fenitrothion [95%; Tyeng Long Inc.], and pirimiphos-methyl [90%; Nan Sing Chemical Meg. Co., Ltd.]), propoxur (97%; Tyeng Long Inc.), fipronil (95%; Aerolead International Ltd.), and imidacloprid (95%; Aerolead International Ltd.) [8]. Each insecticide was diluted to 1% with acetone for the discriminating dose or concentration prepared. The discriminating dose or concentration was twice the 99% lethal dose or concentration of susceptible strains established in 2018 (house flies and cockroaches) and 2020 (mosquitoes) (Table 1).

**Table 1:** Discriminating doses or concentrations of ten insecticides for five insects established in 2018–2020

Insecticides,	*Musca domestica* (ηg/female)	*Blattella germanica* (µg/male)	*Periplaneta americana* (µg/male)	*Aedes albopictus* (µg/bottle)	*Aedes aegypti* (µg/bottle)
Cypermethrin	67.60	29.22	6.80	3.24	0.82
Tetramethrin	13.80	5643.00	4153.00	171.01	31.61
Permethrin	37.80	23.20	19.14	51.03	29.53
Deltamethrin	0.96	22.22	0.96	7.25	0.84
Chlorpyrifos	666.00	35.90	44.76	0.80	0.95
Fenitrothion	544.00	10.96	49.92	0.14	0.05
Pirimiphos-methyl	57.60	87.14	83.66	8.85	0.86
Propoxur	161.20	28.40	8.82	6.38	7.47
Fipronil	37.00	0.74	47.90	0.96	0.96
Imidacloprid	588.00	39.16	36.76	0.84	1.02

### Rapid bioassays

#### House flies

Rapid bioassays were performed using the topical method [8]. Adult female house flies (age, 5–7 days) were anesthetized with carbon dioxide and then temporarily placed in an insect anesthesia device. Each insect was topically treated using a microapplicator (Type MSN-100 microsyringe; Terumo Taiwan Medical Co., Ltd, New Taipei City). For each insecticide, 1 μl of the discriminating dose of each insecticide dissolved in acetone was dropped onto the mesonotum of the house flies. The control flies were treated with acetone only. A total of 20 house flies were used per assay. After topical application, the flies were placed in plastic cylindrical tubes (diameter, 7 cm; length, 12 cm) covered with plastic gauze (80 mesh); then, the tubes were secured on both sides with rubber bands. Cotton wetted in 10% sugar syrup was placed on the tubes for feeding. Each assay was performed in triplicate. The final mortality was assessed after 24 h of insecticide exposure, and the house flies were assumed to be dead if they were ataxic.

#### Cockroaches

Cockroaches were anesthetized with carbon dioxide. Then, a 2-µl discriminating dose of each insecticide was dropped onto the first and second abdominal segments of the cockroaches. Twenty male German cockroaches and ten male American cockroaches were tested each time. All experiments were repeated in triplicate. After treatment, a fluon-coated cylindrical acrylic insect detection device (height, 15 cm) was placed on the wall; food and water were provided ad libitum. Mortality was observed after 24 h. The control group was only treated with acetone.

#### Mosquitoes

The bottles used for the bioassay were coated inside with the discriminating concentration of the insecticide under evaluation. As Table 1 shows, the discriminating concentration was a predetermined amount of insecticide per bottle. One milliliter discriminating concentration of each insecticide was added to a 250-ml Wheaton bottle in accordance with the bottle bioassay method described by the Centers for Disease Control and Prevention (CDC) [26]. The insecticide was evenly distributed on the inner wall of the glass bottle by using a film-rolling machine. Each glass bottle was wrapped in aluminum foil to prevent ultraviolet (sunlight) exposure, closed with tightly fitting caps, and then stored at 4 °C–6 °C for subsequent experiments. Next, 20 adult female mosquitoes (age, 3–5 days; unfed) were aspirated into the glass bottle by using a sucking tube. Mortality was recorded every 15 min until all tested insects died or for a cumulative observation period of 2 h. Each insect was tested three times. The control group was treated with only acetone. The diagnostic time was 30 min. Mortality was defined as the inability of the mosquitoes to stand after being tested [26, 27].

### Data analysis

(i) The corrected mortality rate (%) was calculated using Abbott’s formula (1925) [28]: Abbott’s corrected mortality rate (%) = [(X − Y)/X] × 100, where X represents the survival rate of the control group and Y represents the survival rate of the experimental group. This formula is not applicable when the mortality rate in the control group exceeds 20%.

(ii) Insecticide resistance was evaluated on the basis of mortality rate. Mortality rates of 98–100%, 90–97%, and < 90% indicate no insecticide resistance, possible insecticide resistance, and insecticide resistance, respectively [26, 29].

(iii) Cross-resistance occurs when an insect develops resistance against a certain insecticide and another insecticide that it has not been exposed to. Insecticides with the same mechanism of action typically lead to cross-resistance. Multiple resistance is defined as the development of resistance by an insect to two or more insecticides through multiple mechanisms [30].

## Results

Table 2 presents the results of the rapid bioassays of 10 commonly used insecticides against *M. domestica*. The susceptible strains of *M. domestica* and five wild strains collected from various regions in Yunlin County were tested. All five wild strains are resistant to all 10 insecticides. They exhibit cross-resistance to four pyrethroid insecticides, namely, cypermethrin, tetramethrin, permethrin, and deltamethrin, and three OP insecticides, namely, chlorpyrifos, fenitrothion, and pirimiphos-methyl. Moreover, the wild strains exhibit multiple resistance to pyrethroid, OP, and carbamate insecticides (propoxur) as well as other insecticides such as fipronil and imidacloprid.

**Table 2:** Results of the rapid bioassays of 10 commonly used insecticides against *Musca domestica*

Insecticides	Mortality rates^*^					
	Susceptible	Yuanlin (East)	Erlin (West)	Zhutang (South)	Changhua (North)	Xihu (Central)
Cypermethrin	100% (–)	43% (+)	31% (+)	58% (+)	70% (+)	66% (+)
Tetramethrin	100% (–)	13% (+)	3% (+)	21% (+)	5% (+)	1% (+)
Permethrin	100% (–)	1% (+)	5% (+)	1% (+)	3% (+)	11% (+)
Deltamethrin	100% (–)	15% (+)	13% (+)	16% (+)	15% (+)	16% (+)
Chlorpyrifos	100% (–)	11% (+)	1% (+)	10% (+)	60% (+)	15% (+)
Fenitrothion	100% (–)	6% (+)	1% (+)	11% (+)	13% (+)	13% (+)
Pirimiphos-methyl	100% (–)	1% (+)	5% (+)	3% (+)	10% (+)	6% (+)
Propoxur	100% (–)	5% (+)	8% (+)	3% (+)	13% (+)	6% (+)
Fipronil	100% (–)	50% (+)	51% (+)	53% (+)	65% (+)	68% (+)
Imidacloprid	100% (–)	15% (+)	3% (+)	5% (+)	8% (+)	6% (+)

^*^Mortality rates of 98–100%, 90–97%, and < 90% within 24 h indicate no resistance (–), possible resistance (±), and resistance (+), respectively

The susceptible strains of *B. germanica* and* P. americana* and their five wild strains collected from various regions in Kaohsiung City were tested. Tables 3 and 4 present the results of the rapid bioassays of 10 commonly used insecticides against *B. germanica* and *P. americana*, respectively. The German cockroach strain collected from Qianzhen District is resistant to permethrin. However, the susceptible strains and all five wild strains of both German and American cockroaches exhibit no resistance to any of other insecticides.

**Table 3:** Results of the rapid bioassays of 10 commonly used insecticides against *Blattella germanica*

Insecticides	Mortality rates^*^					
	Susceptible	Daliao (East)	Gushan (West)	Qianzhen (South)	Nanzi (North)	Sanmin (Central)
Cypermethrin	100% (–)	100% (–)	100% (–)	100% (–)	100% (–)	100% (–)
Tetramethrin	100% (–)	100% (–)	100% (–)	100% (–)	100% (–)	100% (–)
Permethrin	100% (–)	98% (–)	100% (–)	83% (+)	100% (–)	100% (–)
Deltamethrin	100% (–)	100% (–)	100% (–)	100% (–)	100% (–)	100% (–)
Chlorpyrifos	100% (–)	100% (–)	100% (–)	100% (–)	100% (–)	100% (–)
Fenitrothion	100% (–)	100% (–)	100% (–)	100% (–)	100% (–)	100% (–)
Pirimiphos-methyl	100% (–)	100% (–)	100% (–)	100% (–)	100% (–)	100% (–)
Propoxur	100% (–)	100% (–)	100% (–)	100% (–)	100% (–)	100% (–)
Fipronil	100% (–)	100% (–)	100% (–)	98% (–)	100% (–)	100% (–)
Imidacloprid	100% (–)	100% (–)	100% (–)	100% (–)	100% (–)	100% (–)

^*^Mortality rates of 98–100%, 90–97%, and < 90% within 24 h indicate no resistance (–), possible resistance (±), and resistance (+), respectively

**Table 4:** Results of the rapid bioassays of 10 commonly used insecticides against *Periplaneta americana*

Insecticides	Mortality rates^*^					
	Susceptible	Daliao (East)	Gushan (West)	Qianzhen (South)	Nanzi (North)	Sanmin (Central)
Cypermethrin	100% (–)	100% (–)	100% (–)	100% (–)	100% (–)	100% (–)
Tetramethrin	100% (–)	100% (–)	100% (–)	100% (–)	100% (–)	100% (–)
Permethrin	100% (–)	100% (–)	100% (–)	100% (–)	100% (–)	100% (–)
Deltamethrin	100% (–)	100% (–)	100% (–)	100% (–)	100% (–)	100% (–)
Chlorpyrifos	100% (–)	100% (–)	100% (–)	100% (–)	100% (–)	100% (–)
Fenitrothion	100% (–)	100% (–)	100% (–)	100% (–)	100% (–)	100% (–)
Pirimiphos-methyl	100% (–)	100% (–)	100% (–)	100% (–)	100% (–)	100% (–)
Propoxur	100% (–)	100% (–)	100% (–)	100% (–)	100% (–)	100% (–)
Fipronil	100% (–)	100% (–)	100% (–)	98% (–)	100% (–)	100% (–)
Imidacloprid	100% (–)	100% (–)	100% (–)	100% (–)	100% (–)	100% (–)

^*^Mortality rates of 98–100%, 90–97%, and < 90% within 24 h indicate no resistance (–), possible resistance (±), and resistance (+), respectively

The susceptible strains of *Ae. albopictus* and *Ae. aegypti* and their five wild strains collected from various regions in Kaohsiung City were tested. Table 5 presents the results of the rapid bioassays of 10 commonly used insecticides against *Ae. albopictus*. The *Ae. albopictus* strains collected from Gushan District exhibit possible resistance to permethrin, deltamethrin, and fipronil. The strains collected from Qianzhen District exhibit possible resistance to cypermethrin and chlorpyrifos and resistance to pirimiphos-methyl. The strain collected from Sanmin District is possibly resistant to pirimiphos-methyl and imidacloprid. However, the five wild strains of *Ae. albopictus* do not exhibit cross- or multiple resistance to pyrethroids, OPs, carbamates, or other insecticides. Table 6 presents the results of the rapid bioassays of 10 commonly used insecticides against *Ae. aegypti*. All five wild strains exhibit cross-resistance to four pyrethroid insecticides, namely, cypermethrin, tetramethrin, permethrin, and deltamethrin, and three OP insecticides, namely, chlorpyrifos, fenitrothion, and pirimiphos-methyl. These findings suggest that *Ae. aegypti* has developed multiple resistance against pyrethroids, OPs, and other insecticides, such as fipronil and imidacloprid.

**Table 5:** Results of the rapid bioassays of 10 commonly used insecticides against *Aedes albopictus*

Insecticides	Mortality rates^*^					
	Susceptible	Daliao (East)	Gushan (West)	Qianzhen (South)	Nanzi (North)	Sanmin (Central)
Cypermethrin	100% (–)	100% (–)	100% (–)	94% (±)	100% (–)	100% (–)
Tetramethrin	100% (–)	100% (–)	100% (–)	100% (–)	100% (–)	100% (–)
Permethrin	100% (–)	100% (–)	93% (±)	100% (–)	100% (–)	100% (–)
Deltamethrin	100% (–)	100% (–)	94% (±)	100% (–)	100% (–)	100% (–)
Chlorpyrifos	100% (–)	98% (–)	100% (–)	96% (±)	100% (–)	100% (–)
Fenitrothion	100% (–)	100% (–)	100% (–)	100% (–)	100% (–)	100% (–)
Pirimiphos-methyl	100% (–)	98% (–)	100% (–)	21% (+)	100% (–)	91% (±)
Propoxur	100% (–)	100% (–)	100% (–)	100% (–)	100% (–)	100% (–)
Fipronil	100% (–)	100% (–)	91% (±)	100% (–)	100% (–)	100% (–)
Imidacloprid	100% (–)	100% (–)	100% (–)	100% (–)	100% (–)	91% (±)

^*^Mortality rates of 98–100%, 90–97%, and < 90% within 30 min indicate no resistance (–), possible resistance (±), and resistance (+), respectively

**Table 6:** Results of the rapid bioassays of 10 commonly used insecticides against *Aedes aegypti*

Insecticides	Mortality rates^*^					
	Susceptible	Daliao (East)	Gushan (West)	Qianzhen (South)	Nanzi (North)	Sanmin (Central)
Cypermethrin	100% (–)	0% (+)	76% (+)	0% (+)	81% (+)	0% (+)
Tetramethrin	100% (–)	88% (+)	95% (±)	78% (+)	98% (–)	85% (+)
Permethrin	100% (–)	71% (+)	88% (+)	33% (+)	91% (±)	76% (+)
Deltamethrin	100% (–)	78% (+)	21% (+)	45% (+)	83% (+)	8% (+)
Chlorpyrifos	100% (–)	31% (+)	70% (+)	0% (+)	75% (+)	18% (+)
Fenitrothion	100% (–)	73% (+)	100% (–)	0% (+)	100% (–)	0% (+)
Pirimiphos-methyl	100% (–)	76% (+)	16% (+)	13% (+)	91% (±)	58% (+)
Propoxur	100% (–)	100% (–)	100% (–)	100% (–)	100% (–)	100% (–)
Fipronil	100% (–)	88% (+)	91% (±)	76% (+)	100% (–)	91% (±)
Imidacloprid	100% (–)	90% (±)	100% (–)	0% (+)	100% (–)	18% (+)

^*^Mortality rates of 98–100%, 90–97%, and < 90% within 30 min indicate no resistance (–), possible resistance (±), and resistance (+), respectively

## Discussion

Insecticide resistance is a complex phenomenon that is mediated by various mechanisms, such as target site modification, metabolic detoxification, reduced penetration, and behavioral resistance. Target site modification occurs when insects develop mutations in genes encoding the target sites of insecticides, thus reducing their binding affinity [31]. Metabolic detoxification occurs when insects exhibit an upregulated expression of detoxifying enzymes that break down and eliminate insecticides from their bodies. Reduced penetration occurs when insects develop thick cuticles that prevent insecticides from penetrating into their bodies. Behavioral resistance occurs when insects change their feeding or resting behavior to prevent insecticide contact [32].

Because of their high reproductive rate, short life cycle, diverse diet, efficient detoxification system, and ability to develop resistance through gene mutations, house flies can rapidly develop resistance against insecticides. The transmission of resistance genes to the next generation leads to the development of resistant house fly populations. A diverse diet exposes them to different toxins and insecticides, and an efficient detoxification system enables them to rapidly break down and eliminate insecticides from their bodies. In addition, gene mutations can lead to resistance development in house flies. Overall, the combination of these factors increases the severity of insecticide resistance in house flies [33]. In our study, the five wild strains of house flies collected from various regions in Yunlin County were resistant to all 10 commonly used insecticides and exhibit cross-resistance and multiple resistance.

The method used in this study to identify the susceptible strain of mosquitoes was based on detection methods proposed by the CDC and WHO [26, 27, 30]. The established diagnostic concentrations may vary across insects, strains, and insecticides. The diagnostic concentration proposed by the CDC was established considering local strains with relatively low resistance as the standard. The susceptible strain of *Ae. albopictus* used in this study has been bred since 2004 and that of *Ae. aegypti* (Bora Bora strain) has been bred since 1986; this explains why the diagnostic concentrations of various insecticides are lower than those established by the CDC. Our goal is to establish a rapid and easy-to-use comparative method for detecting and monitoring insecticide resistance in various pests. The findings of this study can serve as a basis for selecting appropriate insecticides during disease outbreaks. The diagnostic concentration established using long-term-bred susceptible strains is a rigorous standard for detecting resistance. In Taiwan, persistent insecticide spraying is necessary to effectively control the occasional dengue outbreaks. Therefore, the establishment of Taiwan-specific diagnostic concentrations is necessary for practical applications.

The CDC developed a bottle bioassay as an alternative to the WHO tube test to evaluate the resistance of mosquitoes to insecticides that cannot be impregnated onto filter papers. Compared with insecticide-impregnated papers, the bottle bioassay offers increased flexibility and practicality, enabling the use of additives or surfactants that prevent insecticide compounds from crystallizing and ensuring the uniform coating of bottles. However, because of different study designs and test conditions, the interpretation of the results can be challenging. In addition, the bottle bioassay estimates the time required to knockdown or incapacitate mosquitoes within a 2-h exposure period, whereas the tube test advocates mosquito mortality 24 h after a 1-h exposure as the endpoint for monitoring resistance [29].

The diagnostic time for detection is based on detection methods proposed by the CDC and the WHO [26, 29]. For pyrethroids and OP insecticides, the resistance detection time is 30 min. The mechanisms of action of OPs (chlorpyrifos, fenitrothion, and pirimiphos-methyl), fipronil, and imidacloprid involve oxidation within the organism, which leads to toxicity. Thus, Ops, fipronil, and imidacloprid require longer exposure times than do pyrethroids. We found that when the diagnostic time was set to 1 h, none of the five field strains of *Ae. albopictus* exhibited resistance to OPs (chlorpyrifos, fenitrothion, and pirimiphos-methyl), fipronil, or imidacloprid; however, this finding is not observed when the diagnostic time is set to 30 min. The strains of *Ae. aegypti* collected from Gushan District are resistant to pirimiphos-methyl; those from Qianzhen District are resistant to fenitrothion, pirimiphos-methyl, and imidacloprid; those from Sanmin district are resistant to chlorpyrifos, pirimiphos-methyl, and imidacloprid. When the diagnostic time is set to 2 h, only the strains of *Ae. aegypti* collected from Qianzhen and Sanmin Districts in Kaohsiung City exhibit resistance to fenitrothion and imidacloprid. Therefore, the diagnostic time for OP insecticides should be set to 30 min according to their mechanisms of action or different warning-level standards should be established as required to control dengue outbreaks. In the absence of an outbreak, the diagnostic time of 1 h should be used. When an outbreak occurs, a shorter diagnostic time of 30 min should be adopted to provide a basis for relevant agencies to make decisions in the future.

In this study, rapid bioassays of insecticide resistance in vectors are performed using different doses (or concentrations). The findings reveal that *Ae. aegypti*, *B. germanica*, and *M. domestica* have already developed resistance against several commonly used insecticides. This is a common occurrence, and the severity of insecticide resistance in house flies exacerbates the challenge of vector control. The use of insecticides against which insects have already developed resistance should be temporarily suspended. Insecticides with different effective ingredients should be used in rotation. Moreover, environmental management strategies should be strengthened, such as enhancing environmental hygiene and adopting preventive measures for different pests (e.g. adding screens to doors and windows, adding filters to drainage holes, and properly storing unused food and water) to achieve effective vector management and chemical control.

## Data Availability

The data supporting the findings of this study are available within the article.
